# Evaluating knowledge about HIV and discriminatory attitudes among Pakistani women of reproductive age using 2017–18 Demographic Health Survey data

**DOI:** 10.1038/s41598-023-45117-z

**Published:** 2023-10-19

**Authors:** Sonia Sameen, Maryam Pyar Ali Lakhdir, Syed Iqbal Azam, Nargis Asad

**Affiliations:** 1 Department of Community Health Sciences, Aga Khan University, Stadium Road, P.O Box 3500, Karachi, Pakistan; 2 Department of Psychiatry, Aga Khan University, Karachi, Pakistan

**Keywords:** Diseases, Health care, Medical research

## Abstract

A prominent issue associated with HIV is the stigma around it owing to a lack of awareness. This study aimed to find the association between HIV and AIDS-related knowledge and discriminatory attitudes amongst Pakistani women of reproductive age using the 2017–18 Pakistani Demographic Health Survey (PDHS) data. We analyzed a sample of 3381 Pakistani women of reproductive age using ordinal logistic regression for complex survey data. Two composite variables were created using the HIV module to denote the respondents’ HIV-related knowledge and their attitude toward people living with HIV (PLHIV) and calculated using a scoring method. Additional variables included the respondents’ age, education level, socioeconomic status, residential setting, and HIV testing history. More than half (58.8%) of the respondents presented with a negative attitude toward PLHIV and 64.3% of the respondents had poor knowledge regarding the illness. In the multivariable analysis, knowledge about HIV and level of education reported significant associations with discriminatory attitudes. We concluded that the odds of individuals living in a rural setting and hailing from a low socioeconomic background presenting with a negative attitude towards PLHIV were 2.52 times (95% CI 1.07–5.89) higher as compared to those living in an urban setting from a high socioeconomic background.

## Introduction

### Global burden of HIV: insights and impact

HIV/AIDS is seen as a worldwide health threat^1,2^. According to the UNAIDS, in 2022, there were nearly 39 million individuals living with HIV globally, out of which 1.3 million were newly infected cases. More than half of the people living with HIV (PLHIV) constituted women and girls. Moreover, an estimated 630,000 individuals succumbed to AIDS-related illnesses in 2022^3^. As per the World Health Organization, the human immunodeficiency virus (HIV) can damage or impair immune system function and lead to acquired immunodeficiency syndrome (AIDS), the terminal stage of HIV infection^4^. More than 70% of the world's HIV/AIDS burden falls on Sub-Saharan Africa. If HIV prevention efforts are successful in sub-Saharan Africa, it may reduce the disease's prevalence around the world. In 2021, sub-Saharan Africa experienced a significant share of the global HIV impact, witnessing 65% of all HIV-related deaths and 58% of new HIV infections. Notably, among the affected population, the burden was particularly pronounced for heterosexual women and girls in the region, contributing to 59% of all new HIV cases in the region^5^. In the developed world, the United Kingdom has one of the highest rates of HIV infection, with an estimated 107,800 people currently living with the virus, 6000 having received an initial HIV diagnosis, and 320 having been diagnosed with AIDS^6^. According to recent reports, the number of PLHIV in the Eastern Mediterranean region has increased, and deaths from HIV/AIDS have also increased in this area, making it one of the top two regions in the world. About 80% of all the EMRO, i.e., the WHO Regional Office for the Eastern Mediterranean, cases originate in just five countries, with Pakistan being one of them.

### HIV landscape in Pakistan: current status and challenges

As of 2022, HIV prevalence is estimated to be less than 1% in the general population, with 165 000 HIV-positive people in Pakistan^7^. The relatively low incidence of HIV in the Pakistani population, estimated to be less than 0.1% according to the recent UNIAIDS 2020 progress report, can be attributed to the religious practice of abstaining from non-marital sexual interactions in Pakistan. Predominantly, the provinces of Sindh and Punjab constitute more than 90% of the total PLHIV in Pakistan^8^. Of the 210 000 PLHIV in Pakistan, 50% are in Punjab and 43% in Sindh^9^. Focusing on specific areas, the primary clusters of PLHIV are concentrated in Pakistan's most densely populated cities, including Karachi, Lahore, and Islamabad^8^. Despite a global decline in new HIV cases, Pakistan has witnessed a rise in the number of outbreaks, positioning it as one of the rapidly growing HIV-affected nations in Asia^10^. The 2019 HIV outbreak in Larkana, Sindh, was one such example that contributed substantially to the increase in case numbers after 26,041 people were screened for HIV and 751 people tested positive for the condition^11,12^. It is pertinent to note, however, that only 21% of PLHIV in Pakistan are aware of their status and just 12% are undergoing treatment^13^. Injecting drug users and commercial sex workers are two of the most at-risk populations in Pakistan^14,15^. HIV prevalence among the key population in the National HIV Surveillance Round of 2016–17 reported HIV among injecting drug users to be 16.2%, transgender sex workers to be 18.2%, male sex workers to be 5.0%, and female sex workers to be 4.1%^12^.

### Social stigma and its psychosocial dimensions

Social stigma and discrimination have been one of the most difficult issues experienced by PLHIV^16,17^. Stigma, according to sociologist Erving Goffman, is “an attribute that links a person to an undesirable stereotype, leading other people to reduce the bearer from a whole and usual person to a tainted, discounted one”^18^. Social isolation, increased stress and emotional coping, and diminished access to social and economic resources are just some of the negative outcomes of stigma and discrimination for people living with HIV/AIDS^19,20^. Researchers have found that HIV stigma and discrimination persist in interpersonal, community, and healthcare settings, despite improvements in HIV-related knowledge and treatment. There is substantial evidence that many members of the healthcare industry engage in discriminatory practices, such as HIV testing without patient consent, treatment refusal, and breaching patient confidentiality^21,22^. The effects of stigma and discrimination on the lives of PLHIV are complex, but almost always detrimental. Shame, anxiety, depression, suicidal thoughts, and compromised quality of life are all commonly associated with HIV-related stigma^20,23–25^. The results of several studies have shown that HIV-related stigma reduces access to HIV treatment, lowers utilization of HIV care services, reduces adherence to antiretroviral medication (ART), and ultimately reduces treatment efficacy^25–27^.

### Lack of HIV-related knowledge and its implications

Knowledge about HIV/AIDS is a crucial determinant of discriminatory attitudes against individuals living with HIV^28^. According to Global AIDS Monitoring, young people who have a thorough understanding of HIV prevention and transmission are those who (1) knowing that consistent use of a condom during sexual intercourse and having one uninfected faithful partner can reduce the chance of getting HIV, (2) knowing that a healthy-looking person can have HIV, and (3) rejecting the two most common local misconceptions about transmission/prevention of HIV, i.e., HIV can be transmitted by mosquitoes or by supernatural means^29^. Education and awareness about HIV/AIDS are seen as crucial to reducing people's risk of contracting the virus^30,31^. Knowledge and education about HIV/AIDS help reduce people's vulnerability to the virus, as the prevalence of HIV/AIDS is found to be significantly higher among people who are unaware of the potential routes of transmission^32,33^. Data from recent surveys indicate that much more effort is needed. A 2022 study conducted in Rawalpindi and Islamabad, Pakistan, on over 1300 university students reported a general lack of knowledge about HIV/AIDS transmission methods. Only 1% of the study respondents identified blood transfusions to be a mode of HIV transmission whereas the majority identified commercial sex workers, injection drug users (IDUs), and homosexuals to be at a higher risk of contraction^34^. Only 39% of young women, aged between 15 and 24 years, in eastern and southern Africa and 28% of young women in western and central Africa demonstrated in-depth knowledge of HIV in surveys conducted between 2011 and 2018 as compared to 46% and 31% of young men in the same age group^35^.

### Bearing the brunt: women and the HIV burden

Women continue to experience disproportionately high rates of HIV infection, as highlighted in the 2018 UNAIDS report. According to the report, there are 18.8 million females living with HIV worldwide, and nearly 870,000 new cases are reported annually. Inequalities in social, cultural, economic, and political spheres increase women and girls' susceptibility to HIV^36^. Of all the new infection cases registered globally in 2021, 49% constituted women^37^. In countries South-Asian LMICs, like Pakistan, women's economic autonomy and agency are severely constrained when they are forced to leave school to start families at a young age, as is often the case when they marry at a young age^38,39^. Violence against women has also been identified as a factor that increases their vulnerability to HIV^40^. Similar findings were noted in countries in Africa as well^41^. Young women, especially those living in rural areas, have less HIV-related knowledge as well as limited access to HIV testing and modern contraceptives^14,42^. The current contraceptive prevalence rate in Pakistan is 34%^43^. According to the results of a 2021 study, 29.3% of young women in 51 LMICs reported some knowledge of HIV/AIDS. This number varied widely from 1.0% in Afghanistan in 2015 to 64.9% in Rwanda from 2014 to 2015^44^.

### Study rationale

While there is an increasing corpus of literature on the subject, most research work focusing on HIV and discrimination has concentrated on broad issues associated with HIV stigma. Although research on HIV/AIDS-related knowledge and attitudes among high-risk groups has been conducted, very little data are available on young women's knowledge and attitudes toward PLHIV in Pakistan, and these findings cannot be extrapolated to the entire country^45^. There is a lack of data on how young women in urban and rural Pakistan feel about people living with HIV/AIDS, but the 2017–18 Pakistan DHS survey can help fill that void. Due to the large sample size and overall representativeness of the DHS 2017–18, its results are easily generalizable. We intend to use the available information to assess the attitudes of women of reproductive age toward people living with HIV/AIDS in relation to their own level of knowledge about the disease. The study’s findings will help stakeholders and policymakers understand and address the root causes of prejudice against those living with HIV/AIDS and reallocate funds to health education and awareness campaigns that can help change the public's perception. This shift in views is likely to improve community responses to PLHIV and minimize the stigma associated with HIV/AIDS.

### Study objective

The study used secondary analysis to determine the association between the HIV-related knowledge status and the discriminatory attitude status of Pakistani women aged between 15 and 49 years using the Pakistan Demographic Health Survey 2017–18 data.

## Methods

### Study design

This study is a secondary analysis of data extracted from the Pakistan Demographic and Health Surveys Phase 7.

### Data source

The data utilized in this secondary analysis were extracted from the Pakistan Demographic and Health Surveys Phase 7, commonly referred to as the DHS 7. The Demographic and Health Surveys are comprehensive and nationally representative household surveys conducted in low- and middle-income countries (LMICs) every five years. While the primary focus of the DHS program is on LMICs, exceptions may exist for certain countries. The DHS collects data on population, health, and nutrition indicators from various countries, including Pakistan. The survey instruments consist of four model questionnaires: a household questionnaire, a woman's questionnaire, a man's questionnaire, and a biomarker questionnaire. To ensure representative data, the DHS employs standardized multistage sampling methods, typically two-staged, with weighting applied to the collected data. The DHS data is organized into modules, each designed to categorize the data into a comprehensive array of topics that encompass population, health, and social indicators. Specific modules selected for a DHS survey can vary, tailored to the objectives and contextual needs of the country in question.

This cross-sectional survey used a comprehensive sampling frame based on enumeration blocks (EBs) from the 2017 Population and Housing Census, including regions that were not previously covered, such as Azad Jammu and Kashmir (AJK) and the former Federally Administrated Tribal Areas (FATA), making it nationally representative. A stratified two-stage sampling design, with 16 strata representing urban and rural areas across eight regions, was employed for this survey. In the first stage, 580 clusters (EBs) were selected with a probability proportional to the number of households in each EB. In the second stage, 16,240 households were systematically sampled, with 28 households per cluster. Household selection was centralized to ensure objectivity, and no substitutions or alterations to the pre-selected households were allowed to maintain data integrity.

For this study, data from the HIV Knowledge, Attitudes, and Behavior module of the seventh round of PDHS, conducted in 2017–18, were utilized. The analysis focused on data collected through the women's questionnaire. The study employed primary sampling units, and data weighting was carried out using household weight variables and specified stratification units found within the datasets.

### Study sample

For the purpose of this study, data from the 2017–18 PDHS including all ever-married women aged between 15 and 49 years from the HIV Knowledge, Attitudes, and Behaviour module were used. The HIV module contains data concerning knowledge about HIV/AIDS transmission, prevention, discriminatory attitudes, testing service coverage, behavior, and treatment. The women’s dataset comprising of 15,068 observations was used for this study. The extracted data were then cleaned to fix and remove any incorrect, corrupted, duplicate, incorrectly formatted, and incomplete data. The sample size for the study included observations of 3,381 women of reproductive age, i.e., between 15 and 49 years. All other observations were excluded from the analysis as they reported missing values. Instances of missing data concerning women's attributes primarily resulted from incomplete or absent information related to HIV-related knowledge or attitudes. The category of missing HIV-related data encompassed respondents who had not provided consent to participate, individuals who reported privacy concerns during the interview, and selected interviewees who could not be surveyed due to various other reasons.

### Study measures

#### Outcome variable: discriminatory attitude

Discrimination is an action that is often based on a person's negative attitude toward others^46^. Discrimination involves treating people differently because of assumptions made about them based on their differences. Negative perceptions associated with a certain concept or construct contribute to the oppression of individuals or groups.

For this study, the outcome variable was derived from 6 independent variables from the DHS 2017–2018 designed to measure discriminatory attitudes towards HIV among Pakistani women of reproductive age.

The comprising variables included the respondents' opinions of the following:

1. Children living with HIV should be able to attend school with children who do not have HIV.
2. People hesitate to take an HIV test because they are afraid of how other people will react to a positive result.
3. People talk badly about people with or believed to have HIV.
4. Participants would buy fresh vegetables from a shopkeeper or vendor with HIV.
5. People with HIV or believed to have HIV lose the respect of other people.
6. People fear to get HIV from contact with the saliva of a person with HIV.

The responses to the variables were recorded in the form of a yes or no response. A score was assigned to each variable response with a score of 1 indicating a positive attitude and a score of 0 indicating a negative attitude. A cumulative score was then calculated for each variable ranging from 0 to 6.

The outcome variable was reported in the form of a categorical variable defined on the basis of mean distribution with the final categories defined as:

- 0 = Positive attitude = Score of 4–6
- 1 = Mixed attitude = Score of 3
- 2 = Negative attitude = Score of 0–2

#### Primary exposure variable: knowledge about HIV/AIDS

Knowledge is an important prerequisite for prevention in other areas of HIV transmission. HIV transmission knowledge among HIV-negative individuals is essential to reduce the risk of infection^47,48^. Moreover, HIV transmission knowledge among HIV-positive individuals is necessary to reduce the risk of super-infection, as well as to prevent the spread of infection^48,49^. Knowledge about HIV/AIDS can be determined using an extensive array of factors.

The primary exposure variable was derived using a similar approach as the outcome variable from 11 independent variables designed to assess knowledge about HIV among Pakistani women of reproductive age.

The comprising variables included the respondents' opinions of the following:

1. The participants had heard of HIV/AIDS.
2. There can be a reduced risk of HIV by using condoms.
3. There can be a reduced risk of HIV by having 1 uninfected sex partner who has no other sex partners.
4. A healthy-looking person can have HIV.
5. One can get HIV from mosquito bites.
6. One can get HIV from sharing food with an infected individual.
7. One can get HIV from witchcraft or any other supernatural means.
8. HIV can be transferred from mother to baby during pregnancy.
9. HIV can be transferred from mother to baby during delivery.
10. HIV can be transferred from mother to baby during breastfeeding.
11. Drugs can be given to prevent HIV transmission from mother to baby during pregnancy.

The responses to the variables were recorded in the form of a yes or no response. A score was assigned to each variable response. A score of 1 indicated good knowledge. A score of 0 indicated poor knowledge. A cumulative score was calculated for each variable ranging from 0 to 11.

The primary exposure variable was reported in the form of a categorical variable defined on the basis of mean distribution with the final categories defined as:

- 0 = Good knowledge = Score of 10–11
- 1 = Average knowledge = Score of 9
- 2 = Poor knowledge = Score of 0–8

#### Additional variables

Other variables in the study involved the participants’ socioeconomic and demographic information. These included the age of the respondent recorded in years, their highest level of education categorized as no education, primary education, secondary education, and higher education, their wealth index category, namely low, middle, or high, and their area of residence, i.e., rural or urban. The participants’ responses to ever being tested for HIV were also recorded in the form of a binary response.

### Statistical analysis

Descriptive statistics were run by computing the measures of central tendency and dispersion for quantitative variables, and the frequencies and percentages for categorical variables. Inferential statistics for the study were done using ordinal logistic regression for complex survey data (primary sampling unit, region, and women's individual sample weight variables were used to facilitate complex survey data analysis). For the univariate analysis, the crude odds ratio with a 95% confidence interval was reported using simple ordinal logistic regression for complex survey data. Variables with a p-value of ≤ 0.25 will be included in the multivariable analysis. A stepwise model-building approach using multiple ordinal regression for complex survey data was used for the multivariable analysis and reported the adjusted odds ratio with a 95% confidence interval. Variables with a p-value of ≤ 0.05 were included in the final analysis of the study. Adjusted models were controlled for all the potential confounders. All statistical analysis was performed using STATA v.17 software.

### Ethical considerations

Since this study is a secondary analysis of the DHS 2017–2018, an Ethics Review Committee exemption was acquired from the Aga Khan University (2022-8261-23592).

### Ethics approval and consent to participate

Ethical approval was taken from the Aga Khan University Ethical Review Committee in the form of an exemption (2022-8261-23592). Data set authorization was acquired from the DHS. Routine consent procedures were not applied as this was secondary data analysis.

## Results

The original sample size for the DHS 2017–2018 was 15,068. After data cleaning, a study sample of 3381 complete data observations was analyzed.

Table 1 summarizes the distribution of the background characteristics. The mean reported age for the sample was 33.5 years (SD = 7.5). Most survey respondents (n = 2173, 65.3%) reported having poor knowledge related to HIV/AIDS.

**Table 1:** Distribution of sociodemographic and other factors of ever-married Pakistani women of reproductive age (n = 3381).

Participant characteristic	n (%)
Discriminatory attitude	
Positive attitude	480 (14.2)
Mixed attitude	912 (27.0)
Negative attitude	1989 (58.8)
Knowledge	
Good knowledge	492 (14.5)
Average knowledge	716 (21.2)
Poor knowledge	2173 (64.3)
Age (in years)*	33.5 ± 7.5
Highest level of education	
No education	475 (14.1)
Primary education	502 (14.8)
Secondary education	1192 (35.2)
Higher education	1212 (35.9)
Wealth index combined	
Low	302 (8.9)
Middle	566 (16.8)
High	2513 (74.3)
Type of place of residence	
Rural	1476 (46.7)
Urban	1905 (56.4)
History of testing for HIV	
Yes	231 (6.8)
No	3150 (93.1)

*Denotes the mean and standard deviation of the continuous variable of age.

A nearly equal distribution of participants reported having received secondary (n = 1192, 35.2%) and higher education (n = 1212, 35.9%). A similar pattern was observed for respondents who received no formal education (n = 475, 14.1%) or primary education (n = 502, 14.8%). Nearly 74% (n = 2513) of the respondents were categorized under the high wealth index tertile. More than half of the survey respondents resided in urban areas (n = 1905, 56.4%). The majority of the respondents (n = 3150, 93.1%) reported that they had never been tested for HIV.

Table 2 summarizes the crude odds ratio with a 95% confidence interval using simple ordinal regression.

**Table 2:** Association between knowledge about HIV and other factors with attitude towards HIV using simple ordinal regression reporting the crude odds ratio and 95% confidence interval.

Variable	Crude odds ratio (95% confidence interval)
Knowledge	
Good knowledge	Reference
Average knowledge	1.38 (0.97–1.97)
Poor knowledge	1.26 (0.95–1.68)
Age (in years)	1.01 (0.99–1.03)
Highest level of education	
Higher education	Reference
Secondary education	1.65 (1.33–2.04)
Primary education	1.76 (1.29–2.41)
No education	1.78 (1.24–2.56)
Wealth index combined	
High	Reference
Middle	1.00 (0.73–1.40)
Low	1.23 (0.85–1.78)
History of HIV testing	
Yes	Reference
No	0.78 (0.49–1.23)
Type of place of residence	
Urban	Reference
Rural	1.28 (1.01–1.61)

Table 3 summarizes the adjusted odds ratios and their 95% confidence intervals following multiple ordinal logistic regression for complex survey design. The final variables included in the model were knowledge related to HIV/AIDS and the highest level of education acquired by the respondents. Our study concluded that there was no difference in the odds of women of reproductive age with poor HIV-related knowledge presenting with a negative attitude towards HIV and PLHIV and those harboring good HIV-related knowledge (OR = 1.06; 95% CI 0.78–1.43). We also concluded that women of reproductive age who received no formal education had 1.79 times (95% CI 1.24–2.57) higher odds of presenting with a negative attitude towards HIV and PLHIV as compared to those who received higher education. An interaction term was found between two variables, the wealth index combined and the type of place of residence. The results of this study concluded that the odds of women of reproductive age harboring a discriminatory attitude towards HIV/AIDS and PLHIV and belonging to a low or middle wealth index tertile and living in a rural area is 2.52 times (95% CI 1.91–3.75) the odds of belonging from a high wealth index tertile and living in an urban area when adjusted for other variables.

**Table 3:** Association between knowledge about HIV and other factors with attitude towards HIV using multiple ordinal regression reporting the adjusted odds ratio and 95% confidence interval.

Variable	Adjusted OR (95% CI)
Knowledge	
Good knowledge	Reference
Average knowledge	1.26 (0.88–1.80)
Poor knowledge	1.06 (0.78–1.43)
Highest level of education	
Higher education	Reference
Secondary education	1.65 (1.32–2.05)
Primary education	1.77 (1.30–2.42)
No education	1.79 (1.24–2.57)
Type of area of residence and wealth index	
Urban and high	Reference
Urban and middle	1.00 (0.64–1.58)
Urban and low	0.38 (0.19–0.81)
Rural and high	1.30 (1.00–1.71)
Rural and middle	0.65 (0.34–1.23)
Rural and low	2.52 (1.07–5.89)

## Discussion

HIV-related stigma refers to the public's prejudice and misinformation about those who are HIV-positive. It is the discrimination that results from labeling an individual to be a part of a group that is deemed socially unacceptable^50^. The stigma surrounding HIV/AIDS is often cited as a major contributor to the pandemic, but only a small number of studies have actually shown a correlation between stigma and increased risk behavior^51–53^. The results of our study proved a significant association between discriminatory attitudes and practices towards HIV/AIDS-infected individuals and HIV-related knowledge. A Chinese study on college students noted similar findings regarding the association between discriminatory attitudes and knowledge about HIV, and reported more than half (52%) of its study participants exhibiting a discriminatory attitude towards PLHIV^54,55^. A study conducted in Lebanon also reported similar findings^56^. According to a Ugandan study published in 2021, individuals between the ages of 20 and 24 years with higher levels of education, greater financial stability, and a history of having been tested for HIV/AIDS, were more likely to have in-depth and accurate knowledge of the disease^57^.

More than half of our study's respondents reported poor knowledge related to HIV/AIDS. This finding was consistent with statistics reported by previous studies like a 2019 study from Ghana^58^ and a 2023 study from India^59^. Both Ghana and India, like Pakistan, are LMICs as well. While this is a concerning estimate, it is not surprising because LMICs work with limited resources which leads to poverty, limited access to education, lack of proper healthcare infrastructure and impaired access to healthcare providers, as well as conflicting cultural and religious beliefs and subsequent gender inequality^60,61^. The distribution of participants who received no formal education and primary education was similar. The percentages of respondents who received secondary education and higher education were also similar. Despite having adequate knowledge about HIV and a sound educational background, a notably high proportion of the respondents presented with a negative attitude towards HIV and people infected with HIV/AIDS. This study finding is consistent with another study conducted in Karachi, Pakistan^62,63^. Most of the respondents reported never having been tested for HIV, which is not surprising considering how HIV is an area that many people still hesitate to discuss in public settings. This result aligns with those of previous studies noting reduced HIV testing uptake in the country^14,26,64^.

A highly significant association was found between discriminatory attitudes towards HIV-infected individuals and the highest level of education received by the participants. The findings of our study align with studies done in the past^55,62^. According to UNESCO, education is one of the best HIV prevention tools. Lack of education perpetuates the cycle between HIV/AIDS and poverty, often arising from decisions concerning schooling, child-bearing, finances, and unemployment^65^. A 2015 Uganda study reported a statistically significant, negative association between years of schooling and HIV stigma^66^. The study authors also reported that formal schooling might not, depending on its specific curricular elements, be expected to weaken HIV stigma^66^. In a study published in 2020, researchers in India found that even a brief 2-h educational and skills-based intervention improved participants' knowledge and reduced their negative attitudes toward PLHIV. Regular training and educational workshops may have a lasting impact on the public's understanding of HIV/AIDS and its effects, as well as on the stigma that people living with the virus face on a daily basis^67^.

Age and type of place of residence also revealed a significant association with discriminatory attitudes towards HIV/AIDS in our study. Older individuals are more likely to have a more understanding and accepting attitude and approach toward people with HIV/AIDS as compared to the younger demographic. A part of this may also be the result of lived experiences, i.e., over the years, people get more aware of their surroundings and acquire a more well-rounded and holistic view of concerns like HIV/AIDS. Many studies in the past support our study's findings^55,68,69^. Type of place of residence, i.e., rural or urban residence, is also a noteworthy finding as we can expect people living in rural settings to be less informed about HIV and thus be more discriminatory towards people living with the condition. Previous LMIC-based studies support our study findings^70^. This result contradicts those of a study conducted in Malaysia where urban and suburban residents revealed greater stigma towards HIV-infected individuals as opposed to their rural counterparts^71,72^.

When it comes to health equity and well-being, gender is an important social determinant^73^. Pakistan’s literacy rate stands at 63% as per the Economic Survey 2022. According to the report, the literacy gender gap appears to be closing over time^74,75^ Women’s vulnerability to HIV, especially within the context of LMICs like Pakistan, emphasizes the importance of increasing HIV/AIDS-related knowledge among them, as it offers potential opportunities for the long-term control of the HIV epidemic worldwide^76^. Public health education must empower women to recognize the risk factors, including their susceptibility to contracting HIV from their husbands^77,78^. Empowering and guaranteeing the rights of women to prevent HIV infection, combat stigma, and gain greater access to HIV treatment, care, and support is a central part of the United Nations' HIV infection control strategy^79^. According to a study by Raees et al., the HIV epidemic in Pakistan can be attributed to vertical transmission affecting different groups, including female partners of HIV-positive IDUs, bisexual men, as well as pre-adolescent children. Pakistani society's cultural norms get in the way of providing adequate care for HIV-positive pregnant women and children, increasing the risk of mother-to-child transmission^80,81^. Additionally, capacity building among healthcare providers is important to facilitate basic healthcare education among women and further help them manage their concerns and anxieties^82,83^. In order to invoke change, targeted provision of HIV education and access to HIV screening is needed. HIV prevention services should be integrated with sexual and reproductive health services as part of any effort to reduce the spread of HIV among women. Women’s needs at every stage of their lives should be met, and their human rights should be respected, as part of any integration effort. This includes addressing gender inequality and violence, expanding the availability of safe educational opportunities for girls, and more. Family medicine and maternal child health units can be utilized as windows of opportunity to facilitate women’s education. The healthcare system should be a supportive and safe space where women are educated about their options and free to discuss those options in an honest and open manner with a professional who will treat them with dignity and respect^84^.

### Strengths and limitations

To our knowledge, this study is among the few studies to adopt the use of a composite variable to assess both the primary exposure, i.e., knowledge about HIV/AIDS, and the outcome variable, i.e., discriminatory attitudes towards HIV and people infected with HIV/AIDS. Unlike previous studies that have assessed individual variables from the DHS data, our study uses a scoring system to assess the respondents’ understanding of HIV and the resulting attitude leading to it. Since this study is a secondary analysis of the DHS 2017–2018 data, a factor that contributes to the study’s strength is that it has a large sample size, and it has data from all over the country. That said, this study also has some limitations, most notably the fact that this involves the use of survey data collected cross-sectionally. For this reason, we cannot infer that poor HIV-related knowledge is solely responsible for discriminatory attitudes among women of reproductive age. Moreover, the DHS data reported many missing values resulting in a loss of data which may contribute to discrepancies in accurate result estimates. We can attribute the missing data to the sensitive nature of HIV as a topic of discussion in Pakistan.

## Conclusion

This study makes an important contribution to the literature gap that exists in regard to discriminatory attitudes towards HIV/AIDS and its association with knowledge about HIV/AIDS. Further research is needed to address the issues concerning discrimination towards HIV-infected individuals, especially in the LMIC context. Despite having adequate knowledge about HIV and a good educational background, our study population showed negative views reflected through their discriminatory attitudes towards people living with HIV. Pakistan is a country where the conversation around HIV continues to be relatively controversial, it is about time we shift the focus towards changing the narrative and increasing awareness regarding HIV/AIDS, especially among women.

## Data Availability

The data that support the findings of this study are available to the general public upon permission from the Demographic Health Survey (DHS) website [https://dhsprogram.com]. We acquired a permission letter for access to the database from the DHS program.
